# A Case of Bilateral Globus Pallidus Infarction Onset With Mental Disorder

**DOI:** 10.1155/crnm/4539486

**Published:** 2026-04-13

**Authors:** Yingxia Li, Xiaoming Chen, Tianhong Wang

**Affiliations:** ^1^ Department of Neurology, The First Hospital of Lanzhou University, Gansu, China, lzu.edu.cn

**Keywords:** akinetic mutism, carotid artery dissection, globus pallidus infarction, mental disorder

## Abstract

Bilateral globus pallidus lesions are closely linked to neuropsychiatric manifestations; however, bilateral globus pallidus infarction is frequently misdiagnosed owing to its nonspecific clinical presentation. We report a 45‐year‐old female patient who initially presented with mental disturbance (akinetic mutism, memory impairment, and sleep rhythm disorder) without typical motor deficits. Comprehensive head and neck imaging (including DSA, CTA, and multimodal MRI) combined with extensive laboratory investigations—excluding metabolic, toxic, and inflammatory etiologies—led to the final diagnosis of bilateral symmetric globus pallidus infarction secondary to bilateral internal carotid artery dissection (ICAD). Notably, the patient had a recent neck massage, which may have precipitated ICAD. Following dual antiplatelet therapy and endovascular stent placement for severe left ICA stenosis, the patient achieved significant clinical improvement at 2‐month follow‐up. This case highlights the potential association between neck massage and ICAD‐induced bilateral globus pallidus infarction and emphasizes the importance of multimodal imaging in differentiating vascular mental disorders from primary psychiatric illnesses. Our findings provide new insights into the clinical spectrum and pathogenic mechanisms of this rare condition, aiming to facilitate early recognition and targeted management by clinicians.

## 1. Introduction

Cerebral infarction involving the basal ganglia constitutes a common topography of lacunar ischemic stroke subtype [1–3]. Historically, research has predominantly focused on motor deficits, such as hemiplegia, altered muscle tone, and Parkinsonism. With advancements in neuroimaging techniques, an increasing number of basal ganglia stroke cases characterized by cognitive impairment, emotional disturbance, and behavioral changes have been identified clinically. Among these, psychiatric manifestations arising from bilateral globus pallidus infarction pose considerable diagnostic challenges due to the paucity of reported cases and the absence of distinctive symptoms, which hinder timely and effective diagnosis and management [4].

The globus pallidus, a paired component of the basal ganglia, serves as a central hub in the indirect pathway of the basal ganglia‐thalamocortical circuit. It interacts extensively with the thalamus, prefrontal cortex, limbic system, brainstem, and other regions, integrating multiple neural networks that regulate muscle tone, emotional responses, and cognitive processes. Simultaneous bilateral globus pallidus lesions disrupt these neural circuits beyond the brain’s compensatory capacity for unilateral damage, often resulting in prominent psychiatric symptoms. Recent clinical studies indicate that approximately 12%–18% of the patients with bilateral globus pallidus infarction present with abnormal mental and behavioral manifestations at initial admission [5]. Notably, 30%–40% of these patients are initially misdiagnosed with primary psychiatric disorders, such as schizophrenia or bipolar affective disorder, which frequently delays the optimal treatment window for the underlying cerebrovascular disease.

The present study was conducted in accordance with the Declaration of Helsinki. Written informed consent was obtained from the patient for the publication of the details of their cases. Since this was a noninvasive study, approval from the Institutional Review Board was not needed, according to local regulations.

## 2. Case Presentation

On May 18, 2024, a 45‐year‐old woman was admitted to the Emergency Department of Our Hospital, with the chief complaint of “altered mental status for 10 days, accompanied by one episode of limb convulsions.” Ten days prior to admission, the patient experienced an acute, transient bilateral visual blackout without obvious precipitating factors while riding an electric bicycle. These symptoms resolved completely within approximately 10 min, and she did not seek medical attention at that time. Subsequently, she developed persistent dizziness and mental fogginess, without significant head or neck pain. On the afternoon of the following day, she underwent a massage targeting the forehead, occipital region, and neck at a local parlor, which did not alleviate her dizziness and was not associated with new symptom onset. On the third day, her husband observed that she had become mute, failed to initiate communication, exhibited excessive daytime somnolence, and had diminished facial expressions. Although she could independently perform activities of daily living (ADLs) such as eating and personal hygiene, she no longer engaged in household chores voluntarily. On the fourth day, while attending a social gathering with her husband, she remained unresponsive, sat in a daze without speaking, displayed blunted affect, lacked social initiative, and provided fragmented or occasionally irrelevant responses to questions. She also exhibited notable short‐term memory impairment and bradykinesia. No nausea or vomiting was observed at any time during the entire clinical course. There were no reports of dysphagia, choking, fever, involuntary movements, or urinary/fecal incontinence, but her sleep duration was markedly prolonged. She was initially evaluated at a nearby hospital, where a noncontrast head CT scan revealed no significant abnormalities in brain parenchymal density. A plain head MRI demonstrated “relatively symmetrical long T1 and long T2 signals in the bilateral globus pallidus, with prominent diffusion restriction, hyperintensity on FLAIR, and hypointensity on ADC, accompanied by mild white matter lesions in the bilateral frontal and parietal lobes (Fazekas Grade 1).” She received symptomatic treatment including antidizziness medications and intravenous fluid replacement, with no significant improvement. Two days prior to admission (8 days after symptom onset), the patient experienced an episode of generalized tonic–clonic seizures, characterized by upward deviation of both eyes and loss of consciousness, with concurrent urinary incontinence. The convulsions ceased spontaneously after approximately 1 min, and consciousness gradually returned within 5 min. However, she developed worsening apathy, impaired comprehension and judgment, disorganized thinking, and compromised recent memory, prompting urgent evaluation at the Emergency Department of our hospital. The patient had no significant past medical history, no history of chronic alcohol consumption, no long‐term medication use, and no family history of genetic disorders.

## 3. Physical Examination on Admission

Vital signs: temperature 36.5°C, pulse 54 beats per minute, respiratory rate 20 breaths per minute, and blood pressure 92/59 mmHg. Cardiopulmonary and abdominal examinations were unremarkable. Neurological examination revealed the following: the patient was drowsy, mute, and apathetic, with impaired calculation ability, recent memory, comprehension, and executive function. Orientation to person, place, and time remained relatively intact. Both pupils were equal in size (3.0 mm in diameter), round, and reactive to light. Extraocular movements were full, without gaze deviation or nystagmus. Bilateral forehead wrinkles and nasolabial folds were symmetrical. The tongue protruded midline, and the pharyngeal reflex was slightly diminished. Limb muscle tone was mildly increased, with active tendon reflexes and muscle strength graded 4/5 bilaterally. Meningeal irritation signs were absent, and bilateral Babinski signs were negative. Assessment of the sensory system and coordination was limited due to poor patient cooperation.

## 4. Auxiliary Examinations

### 4.1. Local Hospital Examination (May 14, 2024)

Head MRI plain scan: abnormally distributed signal shadows in the bilateral globus pallidus (significant diffusion restriction); a small amount of white matter lesions in the bilateral frontal and parietal lobes (Fazekas Grade 1) (Figure 1). Dynamic electrocardiogram: sinus rhythm occasionally with arrhythmia, occasional sinus bradycardia (average heart rate 55 beats per minute, maximum heart rate 100 beats per minute), occasional atrial premature beats (5 times), and paroxysmal ST‐T changes.

FIGURE 1Head MRI plain scan (local hospital, May 14, 2024) showing abnormal signals in the bilateral globus pallidus (significant diffusion restriction) and mild white matter lesions.

**Figure:**
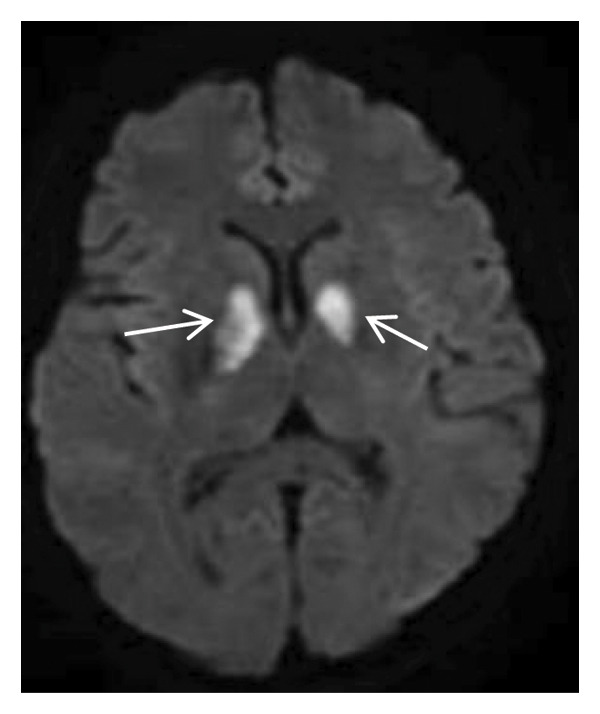
(a)

**Figure:**
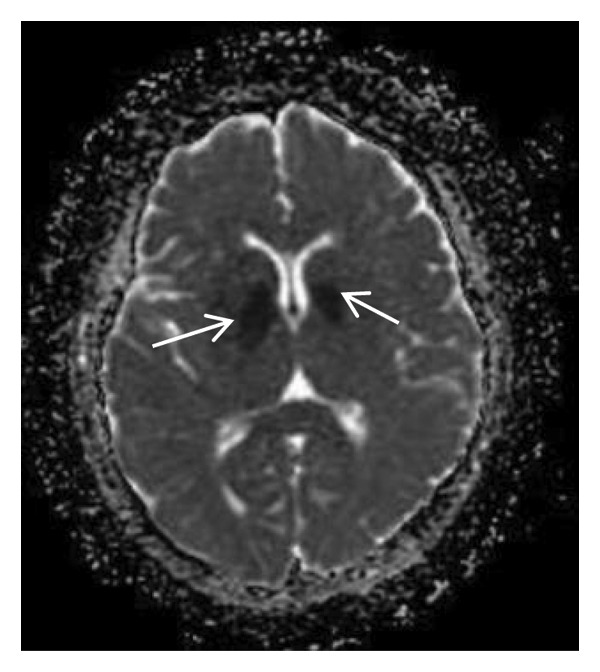
(b)

### 4.2. Further Examinations After Admission

#### 4.2.1. Laboratory Tests

Neutrophil percentage: 80.2% (reference: 40%–75%), neutrophil absolute count: 6.33 × 10^9^/L (reference: 1.8–6.3 × 10^9^/L), lymphocyte percentage: 13.4% (reference: 20%–50%), lymphocyte absolute count: 1.06 × 10^9^/L (reference: 1.1–3.2 × 10^9^/L), eosinophil percentage: 0.3% (reference: 0.4%–8.0%), and all red blood cell and platelet parameters remained within normal reference ranges. Triiodothyronine 1.22 nmol/L (reference range: 1.30–3.10 nmol/L), free triiodothyronine 3.49 pmol/L (reference range: 3.10–6.80 pmol/L); transferrin 1.93 g/L (reference range: 2.00–3.60 g/L); homocysteine 11.70 μmol/L (reference range: 5.00–15.00 μmol/L); antiphospholipid antibodies: IgA < 2.50 APLU/mL, IgG 1.22 GPLU/mL, and IgM 2.42 MPLU/mL (reference range for all: 0–10 APLU/GPLU/MPLU/mL); vitamin B1 (mass spectrometry) 2.65 ng/mL (reference range: 2.50–7.00 ng/mL), ceruloplasmin, immunoglobulin, complement (C3 and C4), erythrocyte sedimentation rate (ESR), tumor markers (CEA, CA199, and AFP), standard fecal/urine tests, soluble transferrin receptor, folate, vitamin B12, anti‐intrinsic factor antibody, antidouble‐stranded DNA, and antinuclear antibody were all within normal limits. CSF paraneoplastic syndrome autoantibody spectrum (14 items) and pathogenic microorganism DNA/RNA sequencing were negative.

#### 4.2.2. Lumbar Puncture

Initial CSF pressure 105 mmH_2_O and final pressure 60 mmH_2_O; CSF appearance was colorless and slightly turbid without clots; CSF routine: white blood cell count 1 × 10^6^/L, red blood cell count 270 × 10^6^/L, shrunken red blood cells 18%; and CSF biochemistry: protein 0.27 g/L, glucose 3.15 mmol/L, and chloride 123.2 mmol/L. Random blood glucose was 5.52 mmol/L. CSF Cryptococcus neoformans/Gatti capsular antigen, bacterial culture, India ink staining, and acid‐fast staining were all negative.

### 4.3. Imaging Examinations

Thyroid color Doppler ultrasound: no significant anomalies detected. Abdominal and urinary system color Doppler ultrasound: cholesterol deposits observed on the gallbladder wall. Routine echocardiography: normal heart structure, blood flow, and left ventricular systolic performance. Bilateral renal artery color Doppler ultrasound: no notable abnormalities found. Thin‐layer plain chest CT scan: scattered small nodules (0.3–0.5 cm) in both upper lobes and the right middle lobe; nodular calcification in the outer basal segment of the left lower lung; minor chronic inflammation in both lower lobes; and slight bilateral pleural effusion. Head MRI plain scan + enhanced scan of intracranial small blood vessels: irregular signals in both globus pallida; white matter hyperintensity (Fazekas Grade 2); developmental venous malformation in the right frontal lobe; and MRA reveals no significant abnormalities in the major intracranial arteries (Figure 2).

FIGURE 2Head MRI plain scan + enhanced scan of intracranial small blood vessels showing abnormal signals in the bilateral globus pallidus, white matter hyperintensity (Fazekas Grade 2), and developmental venous malformation in the right frontal lobe.

**Figure:**
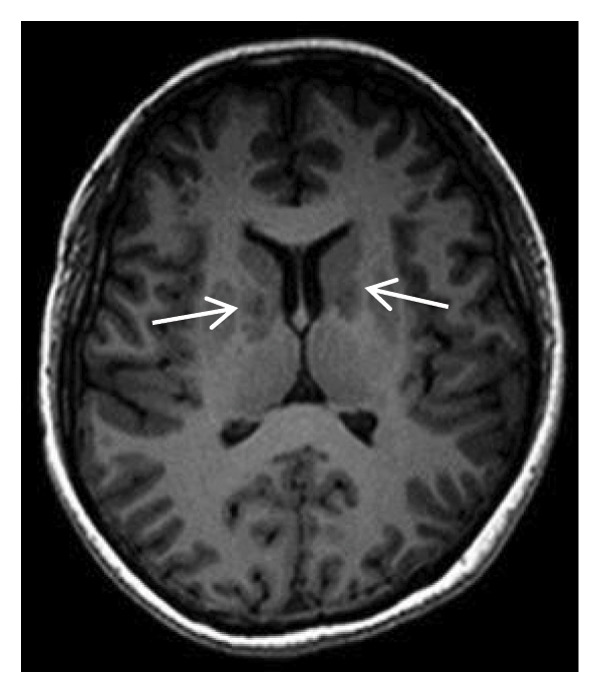
(a)

**Figure:**
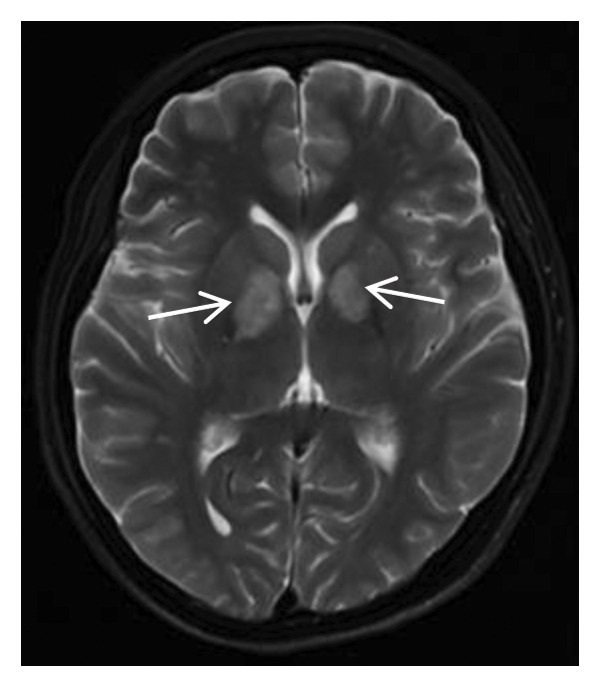
(b)

**Figure:**
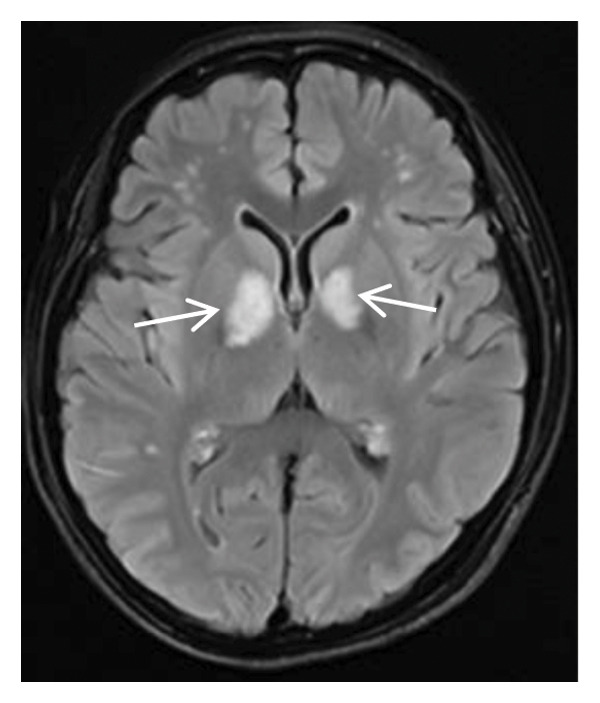
(c)

**Figure:**
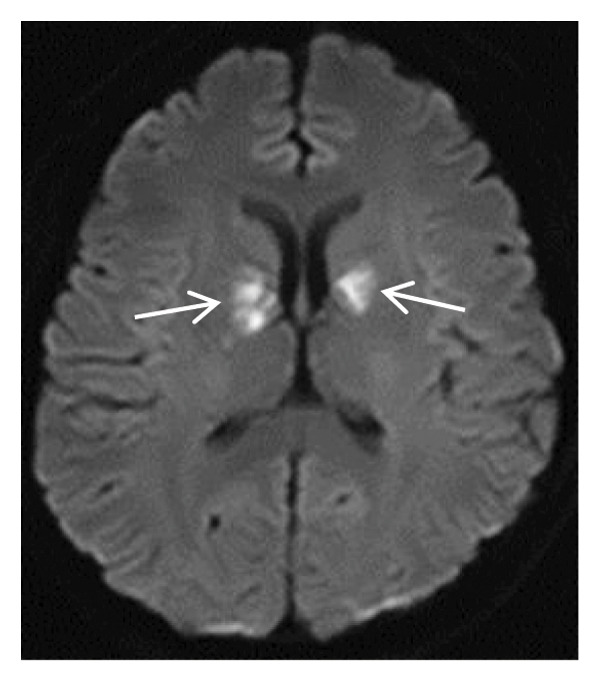
(d)

**Figure:**
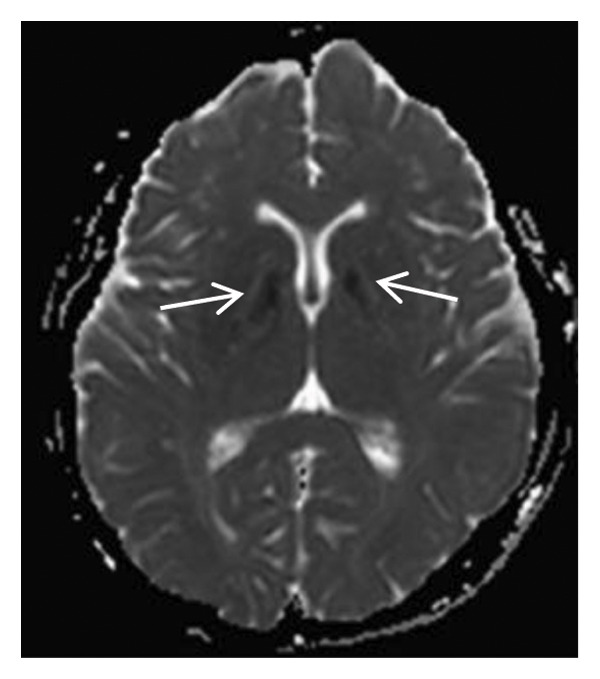
(e)

**Figure:**
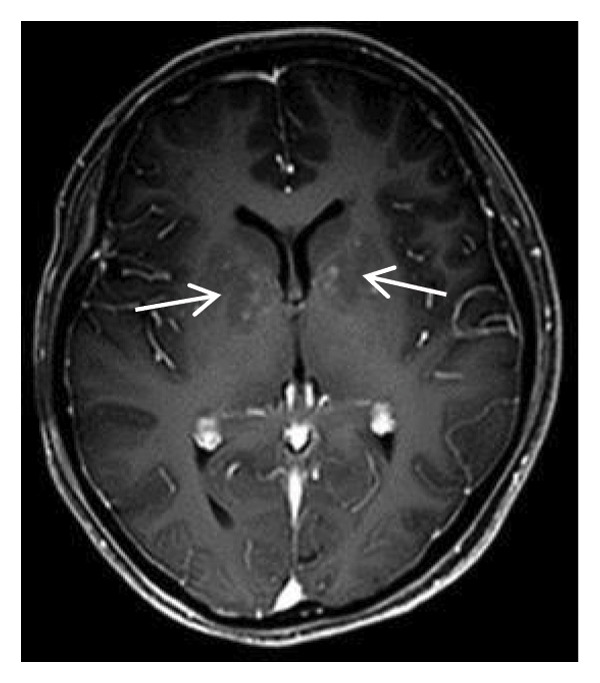
(f)

**Figure:**
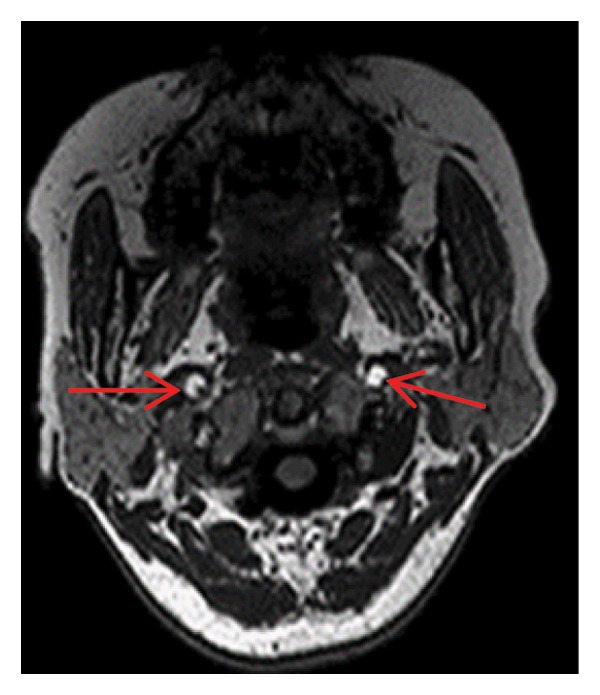
(g)

**Figure:**
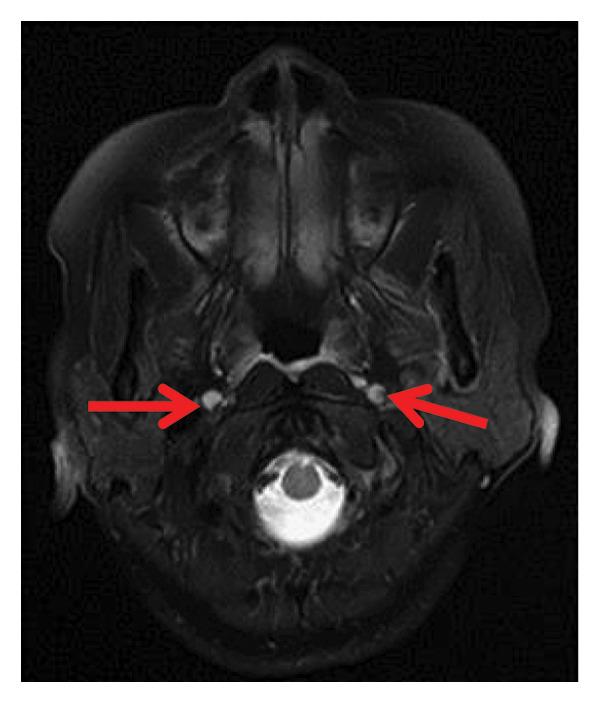
(h)

**Figure:**
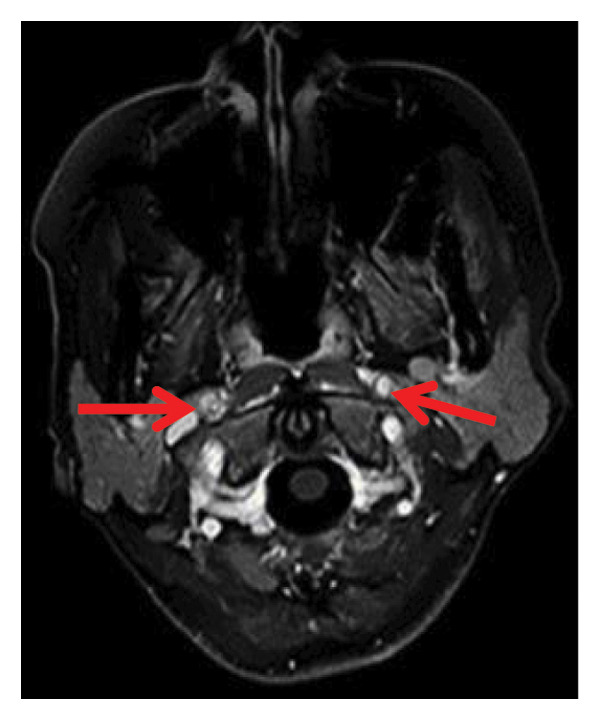
(i)

Head CT plain scan + head and neck CTA + CTP: Patchy areas of slightly reduced density are observed in the right basal ganglia and bilateral centrum semiovale; minor ischemic infarction foci are present in the brain parenchyma; cerebral white matter demyelination is noted; irregular filling defects and significant stenosis are visible in the C1 segment of both internal carotid arteries (CTA); intracranial MTT, CBF, and CBV perfusion appear largely symmetrical; and a patchy shadow with a slightly prolonged TTP is seen in the left centrum semiovale (CTP) (Figure 3). Whole cerebral angiography: Irregular narrowing is identified in the C1 segment of both internal carotid arteries (moderate on the right and severe on the left), suggestive of dissection (Figure 4).

FIGURE 3Head CT plain scan + head and neck CTA + CTP showing patchy low‐density shadows in the basal ganglia, severe stenosis of the bilateral internal carotid artery C1 segment, and localized prolonged TTP in the left centrum semiovale.

**Figure:**
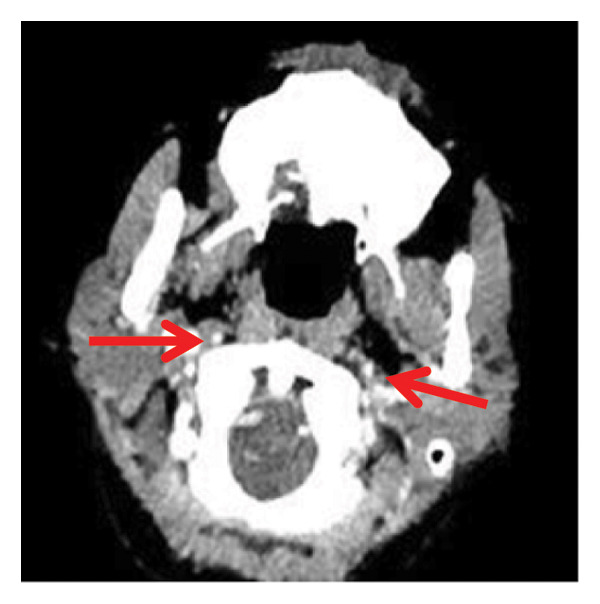
(a)

**Figure:**
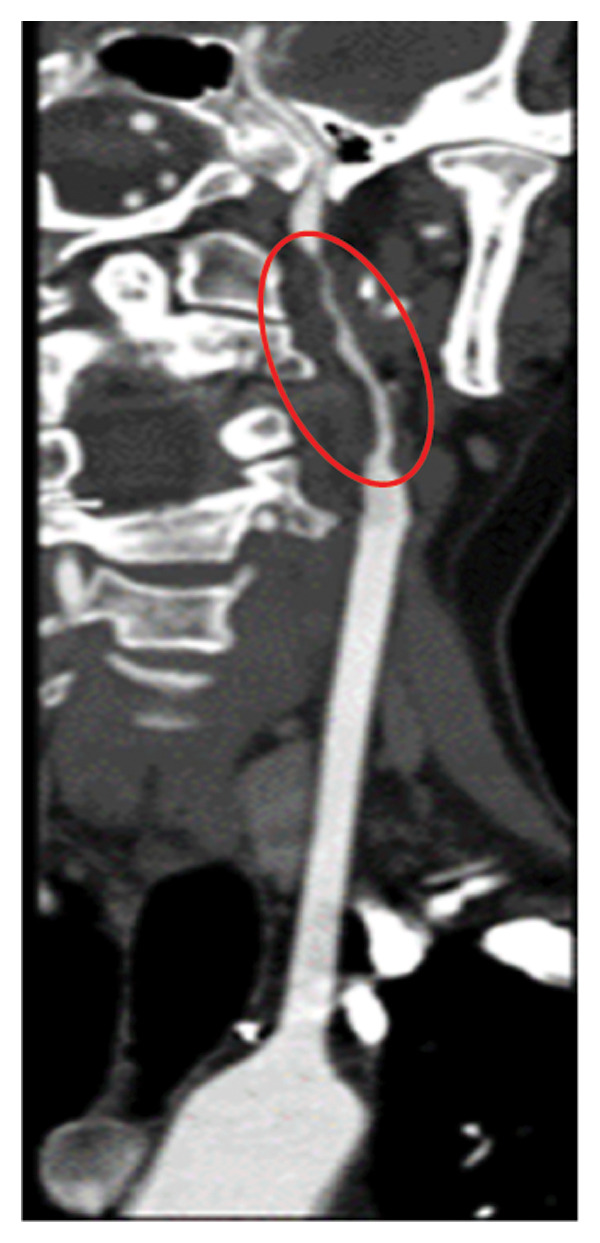
(b)

**Figure:**
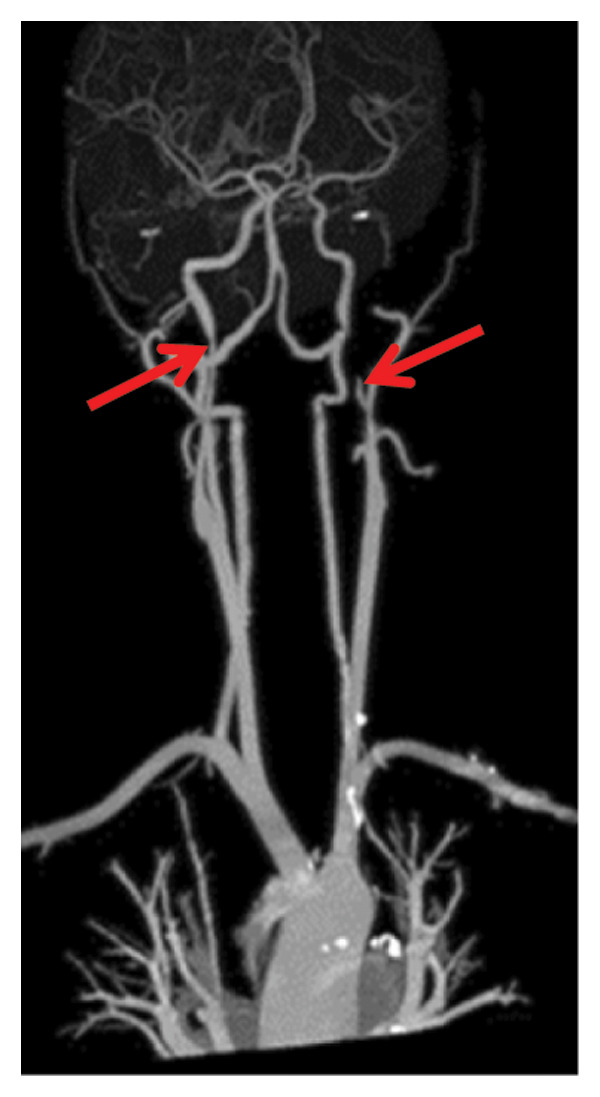
(c)

FIGURE 4Whole cerebral angiography (preoperation) showing irregular stenosis of the bilateral internal carotid artery C1 segment; postoperation image showing stent implantation in the left internal carotid artery.

**Figure:**
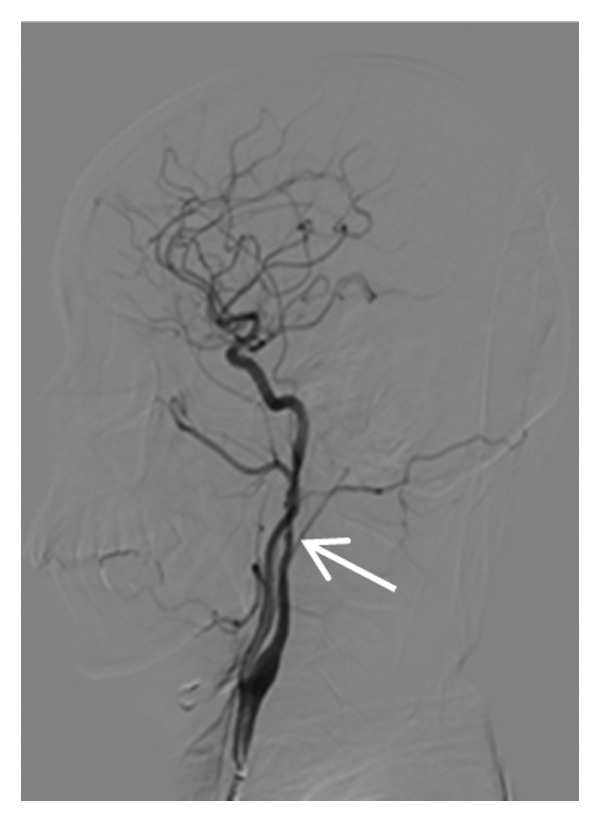
(a)

**Figure:**
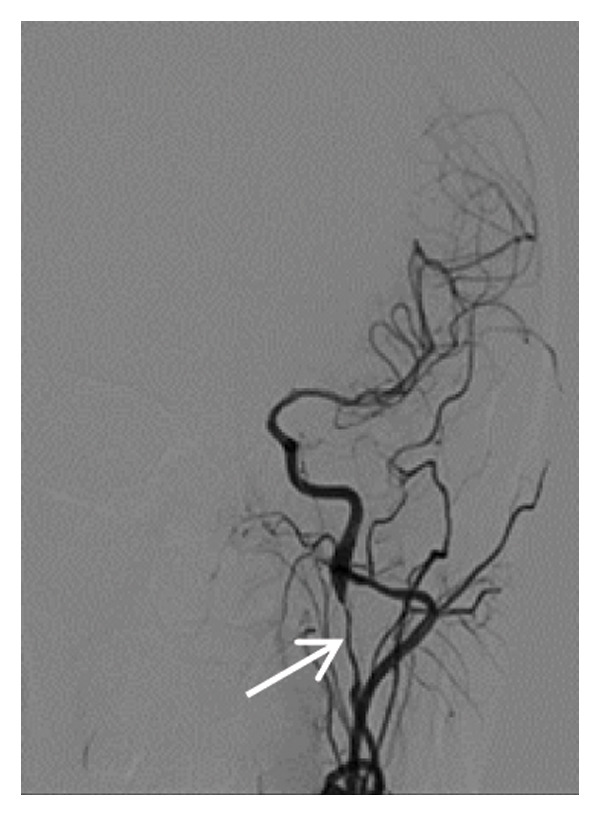
(b)

**Figure:**
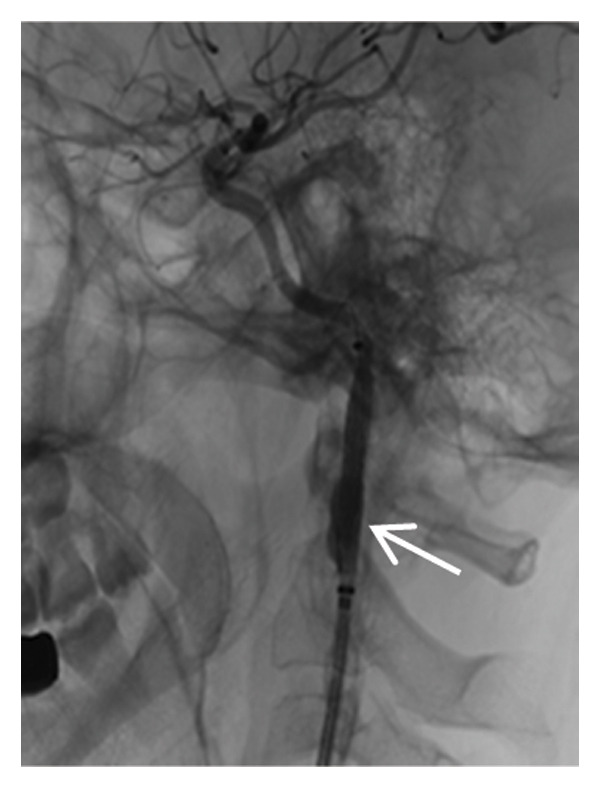
(c)

**Figure:**
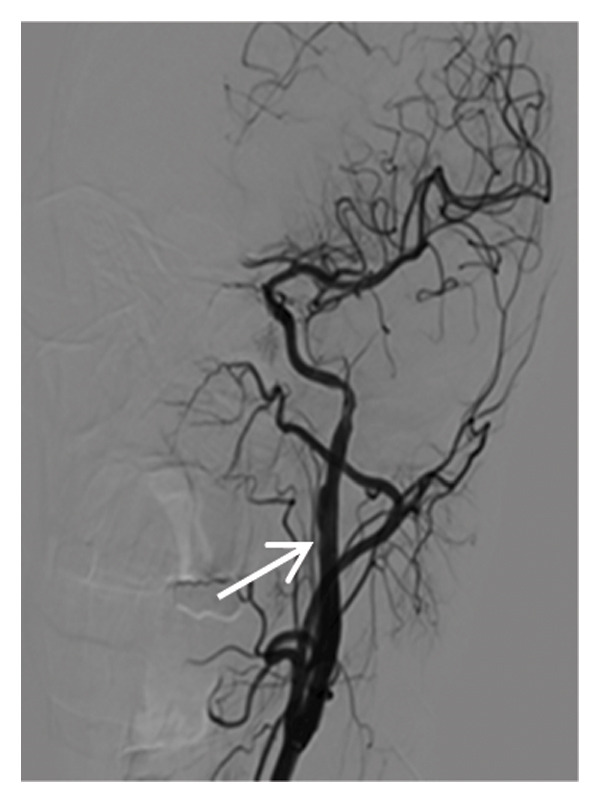
(d)

## 5. Preliminary Diagnosis

Based on the patient’s clinical manifestations, detailed medical history collection, physical examination, and preliminary auxiliary examinations, the following preliminary diagnoses are made: (1) mental disorder of unknown etiology and (2) epileptic convulsion.

## 6. Treatment and Outcome

### 6.1. Treatment

During hospitalization, the patient was prescribed oral medications including aspirin enteric–coated tablets (100 mg once daily), clopidogrel (75 mg once daily), atorvastatin calcium (10 mg once daily), famotidine (20 mg twice daily), and zonisamide (100 mg twice daily). On May 31, 2024, she underwent balloon dilatation angioplasty and stent placement under local anesthesia for severe stenosis secondary to left internal carotid artery dissection (CAD). A Boston Scientific WALLSTENT (9 × 50 mm) was deployed at the stenotic site (Figure 4).

### 6.2. Discharge Status

After a 16‐day hospitalization, the patient was discharged with significant clinical improvement. Her speech returned to near‐normal, her thinking was coherent, and her responses to questions were appropriate. Although she retained mild blunted affect, she could independently ambulate and feed herself.

## 7. Final Diagnosis

(1) Bilateral internal CAD, (2) bilateral globus pallidus infarction (secondary to internal CAD), and (3) epileptic seizure.

## 8. Follow‐Up

### 8.1. Forty Days Postoperatively

The patient’s daily activities were largely unaffected; she independently managed meals and personal hygiene without recurrent seizures. She remained somewhat reserved with limited social initiative but had intact memory and orientation and could perform most household chores. Follow‐up head MRI (plain scan + MRA) showed patchy long T1 and long T2 signals in the bilateral globus pallidus (central hypointensity and peripheral slight hyperintensity on FLAIR, hypointensity on DWI, and hyperintensity on ADC), consistent with the recovery phase of cerebral infarction. The luminal morphology of the C1 segment of bilateral internal carotid arteries was significantly improved; intramural hematoma had resolved, and no residual stenosis was identified. Doppler ultrasound demonstrated normal blood flow velocity and spectral waveform in both internal carotid arteries, with good apposition of the left internal carotid artery stent and unobstructed intrastent blood flow (Figure 5).

FIGURE 5Follow‐up head MRI plain scan + MRA showing signs of cerebral infarction recovery in the bilateral globus pallidus and improved luminal morphology of the bilateral internal carotid artery C1 segment.

**Figure:**
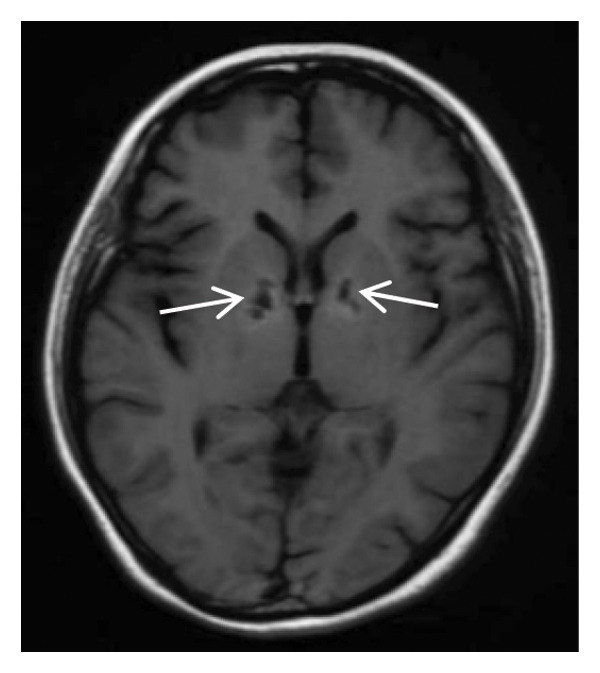
(a)

**Figure:**
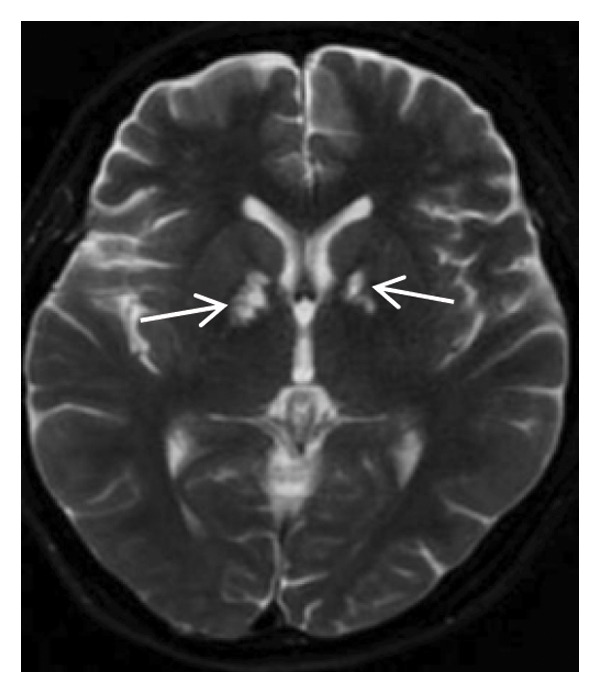
(b)

**Figure:**
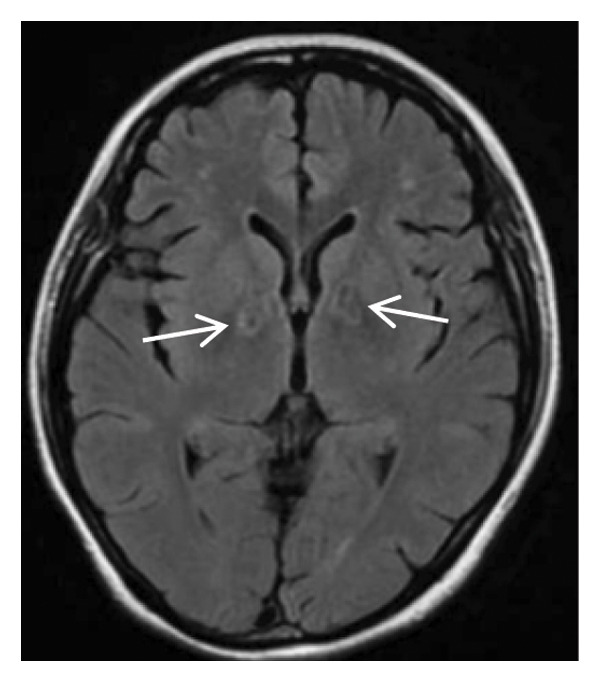
(c)

**Figure:**
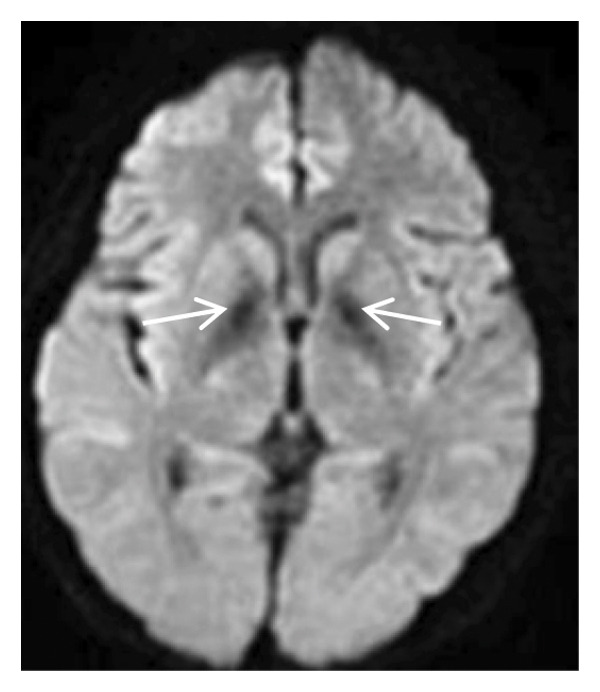
(d)

**Figure:**
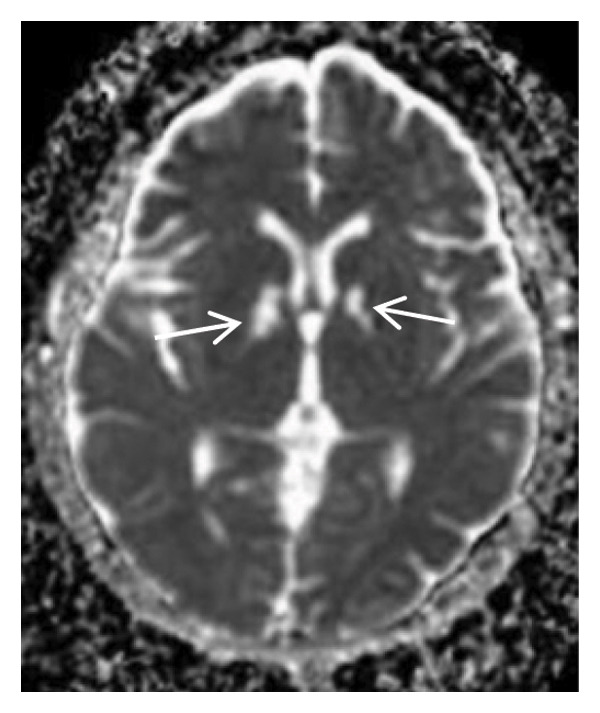
(e)

**Figure:**
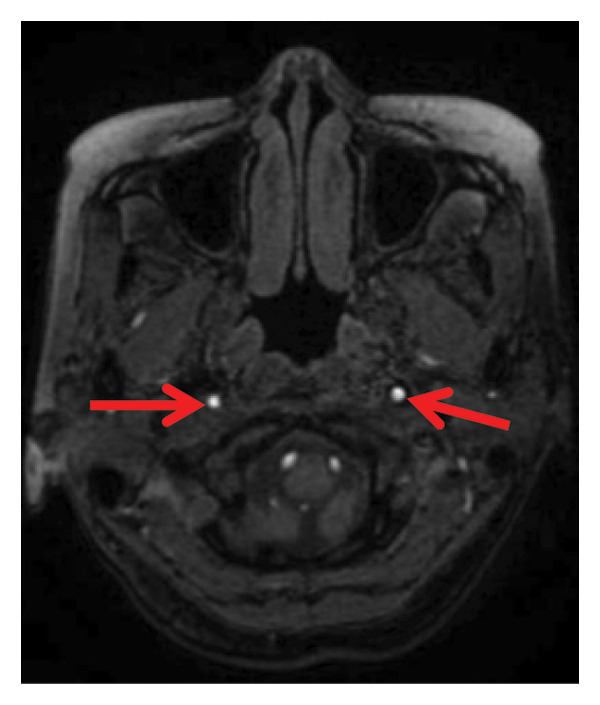
(f)

## 9. Discussion

This case is clinically distinctive for three key reasons: First, the patient presented with isolated mental disturbance (akinetic mutism as the core symptom) without classical motor deficits of basal ganglia infarction, which is rarely reported in previous literature. Second, the underlying etiology was bilateral ICAD potentially triggered by neck massage—a mechanical trauma‐related mechanism that has not been fully elucidated in globus pallidus infarction. Third, the combination of epileptic seizures as a complication of bilateral globus pallidus infarction adds to the clinical heterogeneity of this condition. These features underscore the need for clinicians to consider cerebrovascular etiologies in patients with acute‐onset mental disorders, particularly when accompanied by a history of neck trauma or massage.

As a core component of the basal ganglia, ischemic injury to the globus pallidus and its associated neuropsychiatric manifestations have garnered increasing academic attention. This case report focuses on bilateral globus pallidus infarction presenting mental disorder as the initial symptom. By integrating multidimensional diagnostic modalities—including lumbar puncture, multimodal cranial magnetic resonance imaging, and cerebral angiography—we establish a systematic clinical evaluation framework to clarify the unique symptom progression, imaging characteristics, and key differential diagnostic principles of this condition.

The patient’s primary manifestations included akinetic mutism and sleep rhythm disturbances, with partial symptoms persisting for 2 months posttreatment. Akinetic mutism is characterized by diminished voluntary movement and speech, blunted affect, reduced initiative, and in severe cases, complete loss of behavioral responsiveness and communication with the surrounding environment. It is recognized as a motivational deficit associated with injury or reduced metabolic activity in multiple brain regions [1]. Clinical studies indicate that such cases are frequently misdiagnosed as primary mental disorders due to the absence of distinctive motor symptoms. The underlying neuropsychiatric manifestations are closely linked to three key mechanisms: disruption of the prefrontal lobe‐globus pallidus‐thalamus circuit (governing executive function and leading to behavioral disturbances); dysregulation of neurotransmitters (e.g., dopamine and GABA); and neuroinflammatory responses. This report explores a novel radiomics‐based diagnostic framework and proposes a classification termed the “vascular mental disorder subtype,” providing a theoretical basis for early clinical detection and targeted therapeutic intervention.

The globus pallidus is particularly vulnerable to ischemic and hypoxic injury due to its high metabolic demand. Common etiologies include carbon monoxide poisoning, methanol intoxication, global hypoxic injury following cardiac arrest, basilar artery occlusion, and heroin toxicity. Emerging evidence suggests that head and neck whiplash trauma can induce bilateral CAD, leading to bilateral globus pallidus infarction. Clinical data reveal that mental disorders are the initial symptom in approximately 15%–20% of patients with bilateral globus pallidus infarction, and 42% are initially diagnosed with schizophrenia or affective disorder [6]. Misdiagnosis not only delays the treatment window for cerebrovascular disease but may also exacerbate basal ganglia injury through inappropriate use of antipsychotics. Recent studies have identified a unique neural injury network underlying these manifestations, involving multiple pathological mechanisms: Disruption of the prefrontal lobe‐globus pallidus‐thalamus circuit: this neural pathway regulates executive function and goal‐directed planning. Animal model studies have demonstrated that lesions in this circuit increase error rates in delayed response tasks by 40% in monkeys [7]. Impairment of the limbic system‐globus pallidus circuit: The amygdala and hippocampus regulate emotional responses through projections to the globus pallidus. PET scan data show an 18% increase in amygdalar glucose metabolism in affected patients compared with healthy controls, which correlates with increased anxiety and agitation. Dopaminergic system imbalance: The globus pallidus serves as a key hub in the nigrostriatal dopamine pathway. Postmortem studies have revealed a 35% reduction in D2 receptor density in the globus pallidus and a 20% increase in D1 receptor density in the prefrontal cortex of affected patients, contributing to psychotic symptoms [8]. GABA depletion: The globus pallidus is the primary region housing GABAergic neurons. Bilateral infarction results in a 22% reduction in CSF GABA levels, which disinhibits thalamic activity and leads to cortical overexcitation—an effect positively correlated with hallucinatory experiences [9]. Serotonin (5‐HT) dysfunction: The globus pallidus has indirect connections to the raphe nucleus. Patients exhibit a 15% reduction in platelet 5‐HT uptake rate, which correlates with the severity of depressive symptoms [10]. Glutamate excitotoxicity: bilateral globus pallidus injury induces excessive glutamate release in the thalamocortical projection system. MRI spectroscopy demonstrates a 28% increase in the glutamate/N‐acetyl aspartate ratio in the prefrontal lobe, contributing to cognitive impairment [11]. Genetic and epigenetic factors: the APOE ε4 allele increases the risk of this condition 2.5‐fold compared with noncarriers (38% vs. 15%), exacerbating neural injury by disrupting lipid metabolism. The BDNF Val66Met polymorphism is associated with depressive symptoms, with Met allele carriers exhibiting higher depression scores. Epigenetically, reduced levels of H3K4me3 (a histone modification) in the globus pallidus have been observed, affecting the activity of genes involved in neuroplasticity [12].

The multidimensional pathological mechanisms identified in this case suggest that bilateral globus pallidus infarction–induced mental disorders should be classified as a distinct “vascular mental disorder subtype.” This classification has important clinical implications: unlike primary psychiatric illnesses, this subtype requires urgent vascular evaluation (e.g., DSA or HR‐MRI) and targeted interventions (antithrombotic therapy and endovascular revascularization) to prevent progressive neural injury. Inappropriate use of antipsychotics (which may exacerbate dopamine imbalance) should be avoided until vascular etiologies are ruled out.

CAD represents a significant source of ischemic stroke in younger individuals. Infarctions most frequently occur in the cortical branches of the middle cerebral artery (MCA), whereas damage to the deep perforating branches—such as the lenticulostriate arteries that supply the globus pallidus—is far less common [13, 14]. The underlying cause of CAD remains uncertain. Potential contributing elements encompass arterial disorders, vascular injury from adjacent bony structures, infections, and inherent weaknesses in the vascular wall, which may be triggered by external factors like minor trauma. Research indicates that between 31% and 40% of CAD patients report a history of mechanical trauma before symptom onset, including events such as sneezing, lifting heavy weights, receiving neck massages, or experiencing falls [15, 16]. Globus pallidus infarction secondary to CAD accounts for approximately 5%–8% of all CAD‐related cerebral infarcts, often associated with significant disability and complex neuropsychiatric manifestations [17]. Furthermore, arterial dissection is a relatively uncommon etiology of acute ischemic stroke. A retrospective analysis of 230 patients with acute ischemic stroke revealed that dissection‐related strokes accounted for 7.0% (16 cases), with the vast majority (93.0%) attributed to nondissection causes including large artery atherosclerosis and cardiogenic embolism. In a second analysis of a stroke database encompassing 15,622 patients, only 89 were diagnosed with intracranial arterial dissection, confirming its rare occurrence in this patient population [18, 19]. The underlying mechanisms of globus pallidus infarction in CAD include Hemodynamic compromise: CAD‐induced internal carotid artery (ICA) stenosis reduces perfusion pressure in the M1 segment of the MCA. The lenticulostriate artery, an end artery without collateral circulation, exhibits a direct linear relationship between its perfusion pressure and M1 segment pressure. Beyond 60% stenosis, the hemodynamic reserve of the lenticulostriate artery becomes exhausted. Rabbit model studies have shown that a 50% reduction in ICA blood flow decreases globus pallidus oxygen partial pressure from 38 mmHg to 21 mmHg, approaching the ischemic threshold of 20 mmHg [13, 20]. Steal phenomenon: significant ICA stenosis can trigger a “reverse blood flow” effect, where blood is siphoned from the opposite anterior cerebral artery via the anterior communicating artery. This steal phenomenon further diminishes the blood supply to the lenticulostriate artery [21]. Intramural hematoma–related embolism: a CAD‐induced intramural hematoma discharges microthrombi (fibrin‐platelet microemboli, diameter < 100 μm) that travel into the lenticulostriate artery via the MCA M1 segment [22]. Impaired embolus clearance: reduced cerebral blood flow velocity diminishes microembolus clearance, causing stagnation at the distal end of the lenticulostriate artery. TCD monitoring reveals a 68% positive rate of microembolic signals (MESs) in CAD patients, which correlates positively with the incidence of globus pallidus infarction [23]. Vasospasm triggered by dissection: mechanical irritation of an intramural hematoma or the secretion of inflammatory agents (such as thromboxane A2) leads to constriction in the MCA M1 segment or lenticulostriate arteries. According to HR‐MRI findings, 35% of the patients exhibit lenticulostriate artery trunk spasm, where the diameter narrows to less than 50% of the normal size [24]. Endothelial function impairment: CAD prompts apoptosis in vascular endothelial cells, diminishing nitric oxide (NO) production. Serum endothelin‐1 (ET‐1) concentrations rise by 2.3 times and show a positive association with infarct volume [25].

The etiology of bilateral internal CAD in this patient remains unclear though it may be associated with neck massage. CAD‐induced luminal stenosis compromised cerebral perfusion, ultimately leading to bilateral, relatively symmetrical globus pallidus infarction. The clinical manifestations—including akinetic mutism, sleep rhythm disturbances, and epileptic seizures—are rarely reported in previous literature. Given the severe, near‐occlusive stenosis of the left internal carotid artery, endovascular stent placement was performed to restore vascular integrity, combined with dual antiplatelet therapy. Two‐month follow‐up confirmed near‐complete resolution of the intramural hematoma and successful vascular reconstruction.

This study has several inherent limitations that warrant acknowledgment. First, as a single‐case report involving only one patient, our findings are substantially limited in generalizability to the broader population of individuals with bilateral globus pallidus infarction secondary to internal CAD. Second, the patient’s follow‐up duration is relatively short (2 months after symptom onset and 40 days postoperatively), precluding comprehensive evaluation and analysis of long‐term clinical outcomes—including the risk of recurrent vascular events, the persistence of mild neuropsychiatric symptoms, and long‐term functional recovery. Third, despite conducting extensive laboratory and imaging assessments to exclude common metabolic, toxic, inflammatory, and other vascular etiologies, the potential influence of unrecognized rare etiologies on the onset and progression of the patient’s condition cannot be entirely ruled out.

Based on these limitations and clinical insights, key future research directions are proposed: (1) large‐scale prospective cohort studies are needed to systematically characterize the clinical phenotypes, risk factors, neuroimaging signatures, and prognostic determinants of ICAD‐related basal ganglia infarction, addressing the current gap in data on its natural history and long‐term outcomes. (2) Translational and clinical research should optimize early diagnosis and treatment strategies, including identifying sensitive diagnostic biomarkers, developing personalized antithrombotic regimens, and comparing the long‐term efficacy and safety of endovascular intervention versus conservative management through head‐to‐head trials. (3) Multicenter collaborative studies integrating clinical, molecular, genetic, and advanced imaging data are essential to elucidate the underlying pathological mechanisms (e.g., vascular remodeling, neuroinflammation, and thrombogenesis), laying the groundwork for targeted molecular therapies and advancing precision care for this rare condition.

## 10. Conclusion

Bilateral globus pallidus infarction presenting with mental disorder represents a distinct cerebrovascular entity with unique clinical manifestations. Its pathogenesis involves multilevel disruptions, including neural pathway impairment, neurotransmitter imbalance, and inflammatory cascades. Clinicians should enhance awareness of this condition to facilitate early diagnosis through recognition of acute onset, characteristic imaging findings, and neuropsychological assessments, avoiding misdiagnosis as primary psychiatric illness. Definitive diagnosis—such as in cases of CAD—requires comprehensive evaluation with cerebral angiography, high‐resolution thin‐layer MRI, and diffusion‐weighted imaging (DWI). Management should include personalized antithrombotic therapy based on the underlying mechanism, with endovascular intervention considered for severe stenosis. Future research should focus on developing radiomics‐based predictive models, molecular‐targeted therapies, and individualized antithrombotic strategies to advance precision medicine for this condition.

## Author Contributions

Yingxia Li: drafting of the manuscript. Xiaoming Chen: critical revision of the manuscript for important intellectual content. Tianhong Wang: contributed to the patient’s collections. All authors contributed to the design and interpretation of the study. All authors reviewed the manuscript.

## Funding

This study was supported by the fund of Lanzhou University First Hospital (no. ldyyyn2022‐14).

## Conflicts of Interest

The authors declare no conflicts of interest.

## Data Availability

Data sharing is not applicable to this article as no datasets were generated or analyzed during the current study.
